# The Placenta and its Underestimated Role in Clinical Practice and Research

**DOI:** 10.1055/s-0042-1750156

**Published:** 2022-07-12

**Authors:** Maria Laura Costa

**Affiliations:** 1 Department of Obstetrics and Gynecology, Faculty of Medical Sciences, Universidade Estadual de Campinas, Campinas, SP, Brazil

A few years ago, when I was on my post-doctoral training abroad, on placental biology and immunology, I started to understand the amazing potential within studying the placenta. At the time, my 5-year-old daughter was asked to share with her classmate's information regarding her family and when talking about my background, she said I was a medical doctor. The kids asked: “what kind of doctor?” and without a doubt she answered: “My mom is a placenta doctor.” They further asked what that was, and she finished the subject saying: “something very important.” I believe she was correct. This editorial aims to explain the relevance of the placenta for clinical practice and its research potential. All obstetricians should value the placenta.

It is only fare to start by acknowledging that one of the most significant researchers on the maternal-fetal interface was a Brazilian-British biologist, called Peter Medawar, with special interest in understanding graft rejection and immune tolerance that brilliantly used the placenta as a model for such theories. For his scientific work he was rewarded the 1960 Nobel Prize in Physiology “for discovery of acquired immunological tolerance.” 1 Medawar was born and lived in Brazil during his childhood but decided to move to United Kingdom (UK) and renounced his Brazilian citizenship at the age of 18, when he was obligated to serve in the Brazilian army. Therefore, his academic accomplishments happened in UK, with theories that lasted for long and were essentially: (a) anatomical separation between mother and fetus by the placenta, (b) immaturity of fetal antigens, impairing their ability to produce a maternal immune response, and (c) immunological inertness of the maternal immune system during pregnancy. Nevertheless, we have much evolved and these theories, that guided research in reproductive immunology for decades, have not withstood. For a successful pregnancy, immune function is a complex series of tightly controlled immune modulations, with intense cellular communication in the maternal-fetal interface.

The placenta is the organ that transfers nutrients, gases, electrolytes, antibodies, and other components from the maternal to the fetal environment and simultaneously receives excreta from the fetus. 2 It is also an endocrine organ, with significant hormone production (progesterone, estrogen hormones, human chorionic gonadotropin and somatotropin). The placenta is a discoid-shaped organ measuring 15 to 20 cm diameter, 2 to 3 cm thickness, and around a sixth of the full-term fetal weight, i.e., ∼500 g. It is composed of different cell types, involved in diverse functions that also include adhesion, invasion, vascular remodeling and cell fusion. These characteristics account for similarities between cancer and placental development, since trophoblasts, mimic various malignant cell features. Interesting to note that the placenta invades the adjoining uterus and modulates the maternal immune system, like cancer cells invade neighboring organs, suppressing local immune response. 3

**Fig. 1:**
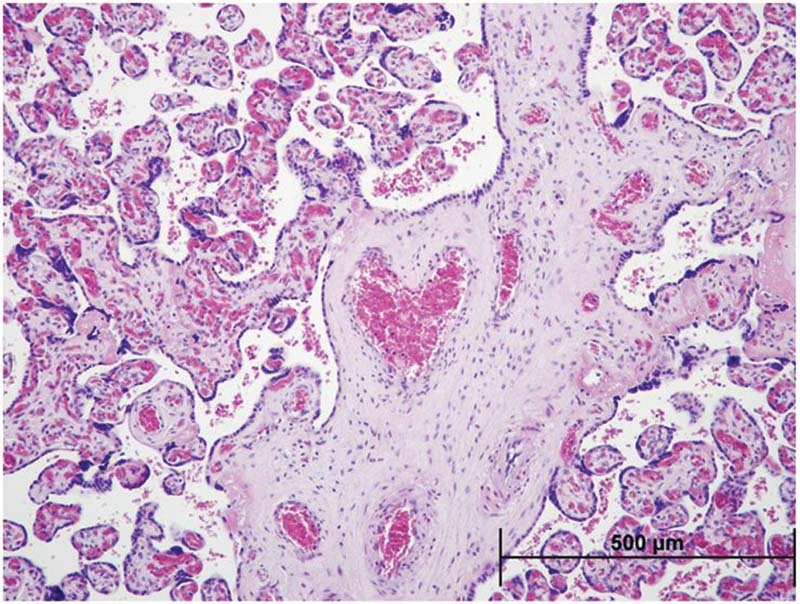
Placental histopathology assessed by H&E staining. A heart shaped vessel within a stem villi in the placenta.

In clinical practice, the placenta is mostly considered a biohazard and discharged after childbirth. 2 However, it is very important to systematically revise its overall aspect and to request a morphological analysis ( Figure 1 ), especially in the presence of adverse maternal and perinatal outcomes. Findings can support or even explain such outcomes. Among the perinatal conditions that should trigger placental examination are stillbirth or perinatal death, fetal growth restriction, hydrops, severe neonatal depression (encephalopathy, seizures), Apgar score less than 3 in the fifth minute, suspected infection, congenital anomalies and thick meconium. Among the conditions associated to childbirth conditions, the indications for placental examination are prematurity (especially <32 weeks), post-maturity or post-date (>42 weeks), oligohydramnios, polyhydramnios, fever or maternal infection, placental abruption. 4

To retrieve valuable information, placental evaluation needs to be performed by a trained pathologist, and preferably using standardized methods and reports. Since 2016, after an expert meeting, an agreement was stablished, called Amsterdam Consensus for the sampling and definitions of macro- and microscopic lesions, broadly classified as: maternal vascular lesions, obstruction of fetal circulation and inflammatory lesions. 5 It is no longer acceptable to have poor reports, that limit findings to a non-helpful sentence such as: “placenta compatible with third trimester” and do not add information to guide clinicians or help in counseling patients.

Congenital infections for example, that represent an important cause of morbidity and mortality worldwide, can benefit from placental analysis. CMV (cytomegalovirus), many times difficult to diagnose during antenatal care, can present with the classical CMV owl's eye inclusions and chronic villitis. Toxoplasmosis shows chronic granulomatous villitis, with immune multinucleated giant cells; parvovirus infection can cause villous edema, erythroblastosis, hofbauer cell hyperplasia and red blood cells with giant viral nuclear inclusions in the pathology evaluation. 6 These findings can answer clinical questions such as: why was this fetus hydropic or why did this case progress to a stillbirth?

Adverse outcomes also rise other concerns, including litigation. The steep increase in litigation in medical practice is a broad and complex subject and urges a support of combined effort between obstetrics, neonatology and pathology, including the examination of the placenta. 7

Some conditions, such Preeclampsia are very studied, with known morphological abnormal findings in the placenta. Preeclampsia, especially when early onset (<34 weeks) is associated to increased maternal vascular malperfusion (MVM), including findings of hypoxia, villous infarctions, and hypoplasia. These findings also support increased risk of recurrence and long term consequences for women, such as increased risk of cardiovascular disease later in life. 8

In research, the placenta has a great potential and is rising as a novel resource for explaining underlying causes of adverse outcomes. Advances in molecular biology, cellular isolation and immune assays have enabled such advances. A clear example of the research relevance of studying the placenta is very recent. During the Zika (ZIKV) pandemic, the severe fetal involvement had everyone question the role of the maternal-fetal barrier. The placenta was shown to be a viral reservoir and a possibility for accurate diagnosis, especially when timely investigation was not performed in the onset of symptoms (that were mostly mild). 9

In Brazil, the Ministry of Health soon recognized the potential of examining the placenta and therefore requested that a piece (one 3.0cm fragment) should be sent for analysis in a certified laboratory, as part of the implemented national protocol for ZIKV care. However, there are very relevant procedures to use placental samples, such as timing between delivery and sampling, method of sampling to allow for representative tissue and mostly the storage, with freezing the material as sampled. This explains why none of the sent biopsies to the official laboratories had positive results on viral investigation, while the same placentas, studied in a research protocol (systematically sampled with 4 fragments), showed high frequency of positivity for ZIKV. Nevertheless, the detection of ZIKV RNA in the placenta does not distinguish between fetal and maternal infection. Thus, questions arise as to possible individual characteristics of immune response or placental function, that still need answers. 9 Mouse models that supported ZIKV replication and trans-placental transmission in pregnant dams were also described in the attempt of facilitating the study of viral pathogenesis, in utero transmission, and testing of therapies and vaccines to prevent congenital malformations. 10

The new challenge is the SARS-CoV-2 infection and its possible impact in placental function. 11 This pandemic presented with very low rates of vertical transmission and mild disease in newborns, however, the involved mechanisms are still not clear and the possible impact of arising Variants of Concern (VOCs) on placental function are under investigation.

Awareness about placental relevance is key to understand major factors underlying maternal and perinatal outcomes. Future advances in imaging and also in testing biomarkers produced by placental cells might change clinical management of disease in the near future.
